# Domestic Correspondence

**Published:** 1890-11

**Authors:** 


### Domestic Correspondence.

---

Odontologists.—*Handsome Dinner at the University Club in Honor of Dr. W. D. Miller, of Berlin.*—The Odontological Society of Cincinnati had the pleasure of entertaining Dr. W. D. Miller, of Berlin, one of the many members of the dental profession who have made the United States so justly celebrated abroad, as the Alma Mater of the new-born science. Like many foremost men in the various walks of professional and political life, Dr. Miller is an Ohio man, born and bred. He is the only American holding a professorship in a German university, namely, the University of Berlin. His researches into the causes of dental caries, demonstrated by exhaustive experiments, have made him an authority upon the germ theory of the etiology of dental caries.

The University Club threw open its doors with cordial welcome.

Among those present to do the honors of the evening were Dr. F. A. Hunter, President; Dr. C. M. Wright, Dr. O. N. Heise, Dr. W. M. Williams, Dr. J. R. Callahan, Dr. J. Taft, Dr. J. S. Cassidy,

Dr. E. G. Betty, Dr. M. H. Fletcher, Dr. A. G. Rose, Dr. H. A. Smith, Dr. F. W. Sage, Dr. J. I. Taylor, Dr. G. Mollenaux, Dr. H. C. Matlack, Dr. R. E. Taylor, Dr. F. Brunning, Dr. C. H. Martin, Dr. Sedgwick, Dr. R. J. Poore, Dr. W. A. Bettman, Dr. J. G. Cameron, Dr. D. W. Roudebush, Dr. H. T. Smith, Dr. J. Leslie.

After the customary toasts had been proposed and answered, all bade the guest God-speed and good-by.

---

To the Editor:

On pages 402 and 403 of your journal (July, 1890) appears a letter signed "A Patient," in which a case is described so definitely that the dental profession will see that the "specialist" referred to is Dr. Kingsley.

The "patient" therein described wishes it understood that, while at a certain time he wrote at suggestion a memorandum of certain incidents connected with his case, he never signed such a communication, nor authorized its publication, and, furthermore, he wishes to say that he is indebted to Dr. Kingsley for an instrument that enabled him to speak perfectly.

This was first accomplished by the use of a flexible or soft velum, and subsequently he made, as an experiment, an obturator with a vulcanite bulb which, after being worn a few days, it was decided to copy in gold, and that instrument was worn with satisfaction for many years.

It would be far from the "patient" to have anything said or done that would cast any reflection upon Dr. Kingsley's great skill or his invaluable service; only those who go through like experiences can fully appreciate the help given in such cases.

---

To the Editor:

In the last issue of the International Dental Journal, owing to a mistake of the reporter, I find myself credited with a most remarkable statement before the meeting of the Maryland State Dental Society, in the report of their proceedings, page 649, as follows: "Now, the success of a dental journal does not depend upon what is published in it, or upon how much matter is accumulated for it, but it rather depends largely on how that journal is supported by the profession at large."

What I did say was, "Now, the success of a dental journal depends not only upon what is published in it, and the amount of matter which is accumulated for it, but it also depends largely upon how that journal is supported," etc.

While the statement, as printed, plainly shows the error on its face, I shall feel extremely obliged if you will kindly give space to the above correction.

Very truly yours,

EDWARD C. KIRK.

---

TO THE EDITOR:

I see in your May "Current News" that Dr. A. H. Thompson finds that bichloride of mercury blackens the teeth. I have had the same experience with it, and find the younger the patient the blacker the tooth turns. It discolors the whole root so that it can be seen through the gum.

Another item: Use gylcerine to counteract the effects of carbolic acid on the mucous membrane of the mouth. It is much pleasanter than vinegar, and fully as potent. Take a pellet of absorbent cotton in the foil-pliers and dip in glycerine and apply, and, presto! the white spot is gone like magic.

Yours truly,

ABIEL ROWEN.

